# Lessons learnt from the implementation of the Covid-19 vaccination programme in the Southwest of England

**DOI:** 10.1371/journal.pone.0309230

**Published:** 2024-08-28

**Authors:** Ilhem Berrou, Laura Hobbs, Sue Jones, Sian Hughes, Hannah Bailey, Sally Quigg, Thomas Manning, Anne Morris

**Affiliations:** 1 School of Health & Social Wellbeing, College of Health, Science and Society, University of the West of England, Bristol, United Kingdom; 2 Science Communication Unit, School of Applied Sciences, College of Health, Science and Society, University of the West of England, Bristol, United Kingdom; 3 Bristol, North Somerset and South Gloucestershire Integrated Vaccination Programme, North Bristol NHS Trust, Bristol, United Kingdom; 4 Insights and Public Engagement, Bristol, North Somerset and South Gloucestershire Vaccination Programme, North Bristol NHS Trust, Bristol, United Kingdom; 5 Business Intelligence (Transformation), NHS Bristol, North Somerset and South Gloucestershire Integrated Care Board, Bristol, United Kingdom; St John’s University, UNITED STATES OF AMERICA

## Abstract

**Background:**

Vaccination remains one of the most successful public health interventions in preventing severe disease and death. The roll-out of Covid-19 vaccination programmes has helped protect billions of people around the world against Covid-19. Most of these programmes have been unprecedented in terms of scale and resources, and have been implemented at times of significant humanitarian crisis. This study aims to outline the lessons learnt from the implementation of a regional Covid-19 vaccination programme. These will help inform emergency preparedness and future crisis management.

**Methods:**

This qualitative study sought to explore the key drivers to the successful implementation of the Covid-19 vaccination programme in a region in the Southwest of England, applying the Normalisation Process Theory lens (NPT) to examine multi-stakeholder perspectives. Data collection involved semi-structured interviews with 75 participants. Document analysis was also used to corroborate the findings emerging from the interviews. Inductive thematic analysis of the data was used to identify the key drivers for the successful implementation of the programme. The NPT lens was then applied to map the themes identified to the domains and constructs of the framework.

**Results:**

Ten key drivers to the successful implementation of the Covid-19 vaccination programme locally were identified, including: the clarity and consistency of the programme’s goal; the diverse representation of stakeholders within the programme leadership team and the mechanisms created by this team to ensure psychological safety, autonomy, operational flexibility and staff empowerment; Communication and data specialists’ input, and collaboration with local communities to maximise the reach of the programme; and allocating funding to tackle health inequalities.

**Conclusions:**

This study highlights the lessons learnt from the implementation of the Covid-19 vaccination programme at a local level, and the mechanisms that can be used in future crises to respond efficiently to the needs of individuals, communities and governments.

## Introduction

The World Health Organisation (WHO)’s Global Covid-19 Vaccination Strategy encourages countries to increase access to Covid-19 vaccines and urges them to work towards vaccinating 100% of their healthcare workforce, 100% of their most vulnerable people, and at least 70% of their population [1]. By October 2022, the UK has achieved vaccine coverage rates of 71% for the 1^st^ dose, 67% for the 2^nd^ dose and 53% for the 3^rd^ dose, with vaccine coverage highest in the oldest age groups [2]. Vaccination programmes are the second most successful public health interventions, after clean water, in preventing severe diseases and reducing deaths [3, 4]. Development, dissemination and deployment of vaccines are key to the success of any vaccination programme [5] and warrant sufficient investment and extensive resources. The total costs of the UK Covid-19 vaccination programme remain uncertain, with an estimate of £11.7 billion [6]. This is in line with other nations including Australia’s costs of $25 billion [7], and the United States of America’s $30 billion investment [8]. Furthermore, back in 2020, it was estimated that vaccinating every adult in England (with 2 Covid-19 doses) would increase the NHS vaccination workload by 740% and would need up to 46,000 staff to deliver the COVID-19 vaccination programme based on a 75% uptake rate [6].

Implementing a national Covid-19 vaccination programme, locally, is complex and can fail if local factors, their interplay, and how they influence or are influenced by the national policy are not considered [9, 10]. Evidence on factors affecting local implementation are scarce, given the overwhelming focus of researchers on policy design and evaluation rather than implementation [11]. Understanding the process and drivers of successful implementation of a national health policy, at a local level, is challenging. Not least because those who design a national policy are often different to those who implement it, but also, because decisions relating to communicating policy aims, resource allocation, managing conflicts and inspiring the necessary change rest with local actors [12].

Another aspect to consider is the Covid-19 pandemic in itself, being a “wicked problem”. Although much has been written on wicked problems since 1973, the pandemic was soon defined as one because: the world did not have complete knowledge of its interdependencies and impacts; it involved multiple actors working in diverse sectors and levels, and because the pandemic was intertwined with other wicked problems (such as health inequalities and a crumbling social care system in England) [13]. This context underpins the complexity of implementing a national health programme, during a global crisis, and the importance of studying the drivers that can lead to the successful local implementation of such policies in such circumstances. Therefore, this study aims to evaluate the local implementation of the national Covid-19 vaccination programme in the Southwest of England, to highlight the mechanisms behind its successful implementation in the region. Our findings identify 10 key mechanisms relating to the actors involved, how they collaborated together, and contexts underpinning their decision making and actions. We rely on the Normalisation Process Theory to understand and articulate these factors to allow researchers, policy makers, public health practitioners and others to replicate, compare and report on the processes and outcomes of local implementation to address the research gap.

The UK was the first country in the world to deploy a national COVID-19 vaccination programme [14]. In England, the national Covid-19 vaccination programme is led by NHS England which coordinates and oversees input from various government departments, Health Security Agency and Office for Health Improvement and Disparities, Department of Health & Social Care, NHS Digital and various other health and care organisations [15]. Input from NHS digital has been particularly valuable in defining vaccination cohorts (with advice from the Joint Committee on Vaccination & Immunisation (JCVI)), setting up and managing national booking systems, and managing secure access and data flow from other organisations [15]. Local healthcare providers are then responsible for administering the Covid-19 vaccines through: Hospital hubs vaccinating NHS staff (and certain groups); community vaccine services vaccinating members of the public in primary care and community settings; and mass vaccination centres vaccinating high volumes of people [15].

Managing vaccine supplies and deployment locally, with operational guidance and overall leadership from NHS England, is the responsibility of the integrated care system (ICS), previously called Clinical Commissioning Groups (CCG). An ICS is a local partnership of organisations (the NHS commissioners and Trusts, GPs, local authorities and community and voluntary sector organisations) that work together to plan and commission health and care services that meet the needs of the local populations [16]. There are 42 ICSs across England, one of which is the Healthier Together Partnership, where this study is based. It covers the areas of Bristol, North Somerset and South Gloucestershire (BNSSG), and serves 1 million people who speak over 90 languages and live in coastal, urban and rural communities [17]. Although the area is mostly affluent, it is also home to some of the most deprived locations in the country, where people in their early 50s have the same level of ill-health as people in their late 60s who live in the least deprived areas [18]. People from Black and other minority ethnic populations make up 10% of the population in the area, and many live in the most deprived locations [18, 19]. By 12^th^ February 2023, a total of 2,650,481 Covid-19 vaccinations have been given. This is the equivalent of every GP registered person in BNSSG having 2.5 doses. Of the total number of vaccinations given, 807,890 were first doses, 779,884 were second doses and 331,449 were seasonal booster doses [20].

The English Covid-19 vaccination programme adopted a top down, centralised approach, especially in the initial phases of deployment [14]. However, as the uncertainties around the impacts and interdependencies of the pandemic started to decline, the programme evolved to allow more input from local teams on implementation priorities and targets to address inequalities in Covid-19 vaccine uptake [14].

While local health inequalities existed long before the pandemic, they became particularly prominent determinants of high risk of severe infection, morbidity and mortality since then. Deprivation, ethnicity and other protected characteristics have been linked to severe Covid-19 infection and death in many countries including the UK [21]; France [22]; Italy [23]; Spain [24] and the USA [25]. These factors also tend to be associated with low uptake of Covid-19 vaccines [26–29]. Adapting vaccination programmes to meet the needs of local populations is key to their successful deployment [30], especially in the midst of an unprecedented public health crisis. Mounier-Jack et al.’s study highlights “tensions” between the national drive for a speedy and efficient deployment of the Covid-19 vaccination programme in England versus tailoring local deployment to the needs of communities that have been underserved for decades and are now reluctant to get vaccinated [14]. The Lack of involvement of local actors in the design of the national programme meant that the programme did not meet the needs of many local communities, driving local implementers to deviating from the national policy and adapting it, and its delivery, to the local context [14]. In their study of a regional implementation of the Covid-19 vaccination programme in France, Meriade et al., highlight that local implementation mechanisms are coordinated across three levels: administrative (local authorities and commissioners e.g. ICBs), organisational (hospitals, GP practices and mass vaccination centres), and operational (healthcare teams and their managers) [31]. The authors further explain that actors at administrative and organisation levels adopted top down implementation approaches (centralised decision making), while actors at operational level adopt more horizontal approaches underpinned by “intersectoral and peer-to-peer collaborations for integrated decision making” [31].

There is a body of evidence examining other high-level factors influencing the successful deployment of vaccination programmes. These include: Transparency of vaccine information to maintain the public’s trust in the vaccines [30]; careful consideration of priority groups [32]; improving (convenience of) access to vaccines [33]; optimising the use of existing infrastructure and expertise [34]; and harnessing digital infrastructure to monitor vaccine uptake, efficacy and safety [15, 32]. However, there is less evidence on how local teams implement Covid-19 vaccination programmes and adapt them to meet the needs of local populations. Insights from the ground are key to empowering local teams to innovate and adapt complex interventions such as the vaccination programme, especially in future emergencies.

The use of implementation theories helps researchers evaluate the deployment of complex interventions’, and provide them with the vocabulary to articulate the mechanisms within the healthcare intervention and the process of embedding it in settings, that influence the outcomes (success or failure) of the intervention. One commonly used implementation theory is the Normalisation Process Theory (NPT) [35]. This theory is particularly useful to use when exploring factors that relate to the actions of individuals and groups (collective action) to integrate an intervention in daily practice [36]. Intervention here refers to the deliberate attempt to introduce new or modified patterns of collective action in a healthcare setting, such as introducing the Covid-19 vaccination programme to increase the uptake of Covid-19 vaccines among the target populations [37]. NPT considers the capability of the intervention i.e. how likely it is to fit into routine practice; the context in which it will be implemented i.e. the setting with its inter- and intra- professional interactions; and the following mechanisms through which individuals and groups contribute to the implementation of the intervention [38]: Coherence, cognitive participation, collective action and reflexive monitoring (explained in the following section).

This paper aims to highlight the mechanisms behind the successful implementation of the Covid-19 vaccination programme in BNSSG, applying the NPT lens to examine multi-stakeholder perspectives. We aim to address the question “what strategies have been useful to increase Covid-19 vaccine uptake and why they were successful”.

## Methods

### Design

This was a qualitative study aiming to explore multiple stakeholders’ experiences of the implementation of the Covid-19 mass vaccination programme in Bristol, North Somerset and South Gloucestershire to identify how the programme was implemented, and how it has led to improved Covid-19 vaccine uptake among diverse populations. A pragmatic paradigm was adopted as this study is part of a wider evaluation of the BNSSG Covid-19 vaccination programme. Semi-structured interviews and document analysis were used to understand the drivers that contributed to the successful delivery of the programme.

### Procedure

We invited 130 potential participants to take part in this study. We carried out semi-structured interviews with 75 participants. We also sought the experiences and perspectives of participants who could not take part in interviews. We emailed them the interview questions, to which 16 participants sent their written responses by email. In addition, document analysis was carried out to validate/ corroborate/ triangulate the findings emerging from the interviews, and provide further understanding of how the programme was designed and implemented. The documents included a range of reports and internal correspondence produced throughout the programme timeline. The interviews were carried out, on zoom or face to face, according to the participant’s preference, between 15 March-6 May 2022 and were audio recorded and transcribed. The participants were asked about their role and experience of delivering the Covid-19 vaccination programme, their perspectives on what worked well about the part of the vaccination programme they were involved with, what worked less well, and what could have been done better. The interviewers attempted to explore the characteristics of local vaccination programme delivery models, whether they worked, for whom and why, the facilitators and challenges to communication and collaboration among the various stakeholders, operating at multiple levels, and the key elements to take forward when the world emerges from the Covid-19 pandemic.

### Participants

The participants included a representative sample of the different people involved in the Covid-19 vaccination programme, at varying times, across the programme delivery points, as well as key programme contributors. These include: Healthcare professionals, healthcare staff, staff working at vaccination clinics, and programme contributors including local authority representatives from Bristol City Council, South Gloucestershire Council and North Somerset Council, Primary Care Network leads, members from voluntary organisations, local healthcare organisations and health care providers, community organisations and pharmacy organisations.

### Theoretical framework

The Normalisation Process Theory (NPT) was used to structure the interpretation of themes identified in the data [35]. This theory has been extensively used to study the individual and organisational factors that influence the implementation of complex healthcare interventions and how these become routine practice [39, 40]. A systematic review and meta-analysis by Kafadar et al. highlights that NPT explains the success of various multidimensional interventions in increasing the uptake of vaccines among individuals and communities [41]. We first performed a standard inductive thematic analysis of the data to identify the key drivers for the successful implementation of the programme, and provide answers to the question “what strategies have been useful to increase Covid-19 vaccine uptake and why they were successful”. We then applied the NPT lens to map the themes identified to the domains and constructs of the framework to highlight the mechanisms that helped implement and “routinise” the vaccination programme within daily practice. In addition to the capability and context of the intervention, NPT considers the following domains [38]:

**Coherence:** Relates to the articulation of the intervention (what the intervention is in simple, clear terms) and whether it is clearly described and understood in the same way by stakeholders interacting with it. It also relates to the collective perception of its purpose, benefits, value added and whether the group implementing the intervention perceives that the aims of intervention align with the overall aims of the organisations and or groups implementing it.

**Cognitive participation:** This domain refers to the group implementing the intervention’ commitment and engagement with the implementation of the intervention. It relates to the willingness of the group to actively participate in the implementation of the intervention, based on their shared conviction of its purpose and benefits. This domain reflects the decisions the group makes to dedicate time, energy and other resources to implement the intervention.

**Collective action:** This refers to the individual and group efforts to implement and normalise the intervention. These efforts will be determined by the impact of the intervention on individual and group’s work routine, the resources needed and/ or available to implement the intervention, and how these efforts align (or not) with what the organisation/ group does.

**Reflexive monitoring:** This domain relates to the group’s appraisal of the intervention once it has been implemented and functioned for some time. It considers the potential of sustained benefits and whether these benefits are realised and are tangible or visible to the group. It also relates to the ways in which individuals/ groups can provide feedback on the intervention and adapt it/ improve it as needed in their setting.

These four domains are continuously influenced by the context in which the intervention is being implemented [37–38].

## Results

Table 1 shows the number of participants and stakeholder representation amongst the study sample. A total of 75 participants were interviewed. Responses (sent by email) from those who agreed to participate in the study but could not attend interviews were included in documents’ analysis. Thematic analysis of the data identified 10 key themes, each representing a key driver to the success of the BNSSG vaccination programme. These themes have been mapped to NPT domains, and are presented in Table 1 in S1 Appendix.

**Table 1:** Number of participants from BNSSG Covid-19 vaccination programme contributors.

Participant’s organisation	Interviewed	Emailed responses
Primary Care Network	19	1
Pharmacy	3	-
Hospital Hub	7	-
Mass Vaccination Centre	15	1
Children Immunisation Providers	3	-
Outreach Team	9	1
Local Authorities	10	6
Programme Senior Leadership	2	6
Volunteers and Community Organisations	7	1
**Total**	**75**	**16**

The following sections explain the key drivers of the successful implementation of the BNSSG Covid-19 vaccination programme, supplemented by the participants quotes.

### Key drivers to the successful implementation of the BNSSG Covid-19 vaccination programme

#### 1. One clear, measurable goal

Most participants stated that the programme goal was simple and clear: To vaccinate as many people as possible, as quickly as possible. There were no sub-goals or detailed explanations of the programme’s aim and objectives. The participants agreed that the simplicity and clarity of the goal of the programme meant little ambiguity in understanding and interpretation of the programme tasks, which ensured the coherence of understanding and articulation of the programme. This is illustrated in the quotes from three different stakeholders:

*“We kept the mission clear and central. Our mission was not detailed…we wanted as many vaccines in as many arms as possible, as quickly as possible. It was as simple as that.”*

*“It was a simple and well-defined goal. Everyone including the patients knew what the goal was… and the task was very simple. It wasn’t complicated with a lot of variation attached to it. It was simply how do we contact people, get them booked in, and get around to the housebound and the care homes. And medicine is not often as simple as that.”*

*“There was so much that came along and changed*, *but the goal never changed*. *The job was to vaccinate people to protect them from Covid*.*”*

It was also reported that the goal was measurable, and allowed all stakeholders to track progress of delivery, celebrate success and improve delivery in geographical areas and patient groups lagging behind. A key lesson for the future is to ensure that the aim or mission of the complex intervention is articulated in in simple words that do not contain any jargon, and are not associated with further explanations or objectives. It is also important that the aim remains consistent throughout the implementation of the intervention.

#### 2. One multi-disciplinary leadership team

A multi-skilled, multi-organisational Clinical Delivery Group (CDG) was set up to oversee the programme delivery. CDG members were primary, community and secondary care clinicians; operational managers; regional representatives, communications experts and Insights and Business Intelligence representatives from the Bristol North Somerset South Gloucestershire Clinical Commissioning Group (now Integrated Care Board (ICB)). The CDG met daily to discuss operational issues of the day, present relevant updates and agree the next steps across a wide range of required actions. This ensured that all partners understood the programme in a similar way, and provided the mechanisms to make the programme routine practice. Videoconferencing platforms such as Microsoft Teams, have enabled the formation of such a diverse and geographically dispersed multi-professional team. This diversity, enabled by digital solutions, was key to the success of the programme.

The CDG provided leadership, a safe space to share ideas and concerns, ensured that the local narrative is understood by all organisations, and granted vaccination teams operational flexibility to innovate to solve problems relating to the design and delivery of vaccination clinics.

*2*.*1*. *Leadership*. The collective leadership of the CDG was constantly visible to the various people and organisations involved in the Covid-19 vaccination programme. The CDG provided clinical and operational guidance and support to all stakeholders throughout the implementation process.

*“We pulled in very quickly Public Health, the local authority, local resilience forums, the local council, health systems, commercial partners… Just pulling those people in very quickly and making them feel like equal partners, not just add-ons at the end. That proved very beneficial because we were able to very quickly mobilise sites, like Ashton Gate Football Stadium and shopping centres etc… so that was really helpful…those things happened quickly because we had the right people in the conversation”.*

*“We were all on that one same page about what needed doing*. *And how we are going to get there*. *That is very unusual within the NHS*. *There was nobody else’s baggage*. *There were completely agnostic people who came together to work to get that goal done*. *That got everybody working hard”*.

This collective leadership of the CDG group was underpinned by a deep understanding of the unprecedented nature of the Covid-19 emergency, and the need for trial and error to identify the best approaches to vaccinating people during this emergency.

*“We went with the whole team approach, working together but being open to the fact that we were all learning as we went”.*

*2*.*2 Safe space to share concerns and be creative*. The CDG daily meetings provided a valuable opportunity for members to feel safe and confident to express their concerns and suggest ideas to solve the operational problems of the vaccination programme.

*“I think that there is a lot to be said for a space where you can feel psychologically safe, and there are high levels of trust… and you’re not fearful of saying something that to you, might sound really silly when you say it”.*

*“We could have honest*, *professional and sometimes challenging conversations*, *but they felt very comfortable*.*”*

The multi-disciplinary nature of the CDG team, and its flat hierarchical structure have been suggested by many participants as key to creating this safe supportive space.

*“We were flexible and agile with a flat structure which enabled quick decision making.“*

*“The importance of pulling together the right team from the start…People talk about being ‘voluntold’ to do the roles*. *The programme attracted and brought in like minds who embraced the open*, *collaborative approach*, *who valued the flat hierarchical structure and can-do attitude and supportive space that it evolved into*.*”*

*2*.*3 Adapting the programme to the local narrative*. The BNSSG vaccination programme relied on the commitment and collaboration of multiple organisations to ensure vaccinating as many people as possible, as quickly as possible. These organisations have traditionally operated independently, and knew little about each other. However, the multi-disciplinarity of the CDG with representation from all stakeholders, and the regular meetings and events they organised to present updates on vaccination efforts across all the local vaccine delivery sites and share learning and good practice helped the organisations learn about each other, improve their confidence in their approaches, and optimise processes and resources to reduce vaccine wastage and maximise the number of people to be vaccinated against Covid-19.

*“Because we have a national commissioning framework, one of the things that certainly worked well was that there was a (local) system-led ambition… And I think that was very good because what that did was it allowed us to share system intelligence, while the national team would always have overarching figures, they didn’t have just the narrative. I think having the (local) system narrative was very helpful because it meant, because of the commissioning framework, you couldn’t learn from what was going on in the mass vaccination centre because they were not part of the commissioning framework that you are working under… or from the PCN (primary care network) side because, again they were not a part… That willingness to create a coming together platform was really, really, very helpful”.*

*“Because what those shared learnings have been able to do is they’ve brought confidence into the way we work*. *They’ve also brought more consistency*, *you know*, *in terms of how people do things and the approach that they take”*.

*2*.*4 Governance to allow operational flexibility*. The national Covid-19 vaccination programme was perceived by the participants as restricting, especially in relation to the strict cohort vaccination management, and its generic format does not account for local variations and needs. The CDG allowed quick decision making and granted flexibility to vaccination teams, under a vigilant clinical governance, to design and deliver their vaccination clinics in the way they see fit to deliver on the programme goal to vaccinate as many people as possible as quickly as possible.

*“We were handed a blueprint from NHS England which those of us who had a lot of experience could see would be very limiting. We were instructed not to change without permission which we were also told would be unlikely. But we did it anyway. It was low risk for high reward, proved to be really effective and has now been used in many other systems. This was mainly around workforce and VC (vaccination centre) structure but also applied to other areas.”*

*“So, if I had a problem or suggestion or idea, within 10 minutes everybody would have said yes or no… and by 10 o’clock I’d be having a chat with x and we’d be working out how to do it”*

*“The CDG gave us operational flexibility which was very important*. *I had to be creative and innovative*, *but the CDG gave us permission to move with both pace and agility*. *Usually*, *if I was commissioning something*, *it would have taken me a year to get that through*, *at all kinds of boards*. *But we got our model through in less than a month*. *That just doesn’t happen when its ‘business as usual’”*.

The CDG operated on the basis that vaccinators on the ground are key to solving logistic issues. Their perspectives were regularly received and discussed through video-conferencing platforms. The CDG provided space for people to suggest solutions, actively listened to these suggestions, and helped the vaccination teams overcome the cumbersome bureaucracy NHS staff usually face when innovating or suggesting novel approaches.

*“If something worked better you did it. For example, we had to get stickers to put on the floor, because everybody kept getting lost on the way to the observation room. And we were all amazed that yes, we could do that. In normal times, I would have had to go through 17 committees and all these people sign off on it, then go out for 8 quotes. But with these stickers it was yes, ok and two days later they arrived. The nurse that suggested it…she says it’s the best place she has ever worked, because they listened.”*

Collective leadership, through a multi-stakeholder team with representation from all organisations involved in the implementation of the complex intervention ensures that these organisations are part of the implementation decision-making process. This then strengthens their commitment to implementing the intervention, and provides a mechanism through which they can appraise and adapt the intervention to ensure its successful implementation.

#### 3. Data-informed decision making

The BNSSG ICB, in partnership with the wider health and social care system, and in conjunction with One Care Ltd, a large GP federation, representing and supporting more than 77 general practices and one million patients in BNSSG, has developed a dataset linking together information across primary care, secondary care, mental health and community services for over 1 million patients in BNSSG. This System-Wide-Dataset (SWD) was launched in August 2019 [42], containing patient activity and attributes from primary care GP practice systems and other national datasets from within and outside the NHS, such as the Indices of Deprivation.

The CDG requested data items to capture dates of seasonal flu vaccination and dates of COVID-19 vaccinations to predict trends of vaccine uptake and/or hesitancy. Locations within vaccination centre data was also used to add accessibility elements to the analysis of vaccine uptake rates. The CDG monitored the proxy vaccine uptake rates via the nationally developing ‘Palantir Foundry’ database and maintained a watching brief on vaccination data flowing from national systems and into GP records, despite the irregularity and unpredictability of data flows.

Early successful uses of the SWD to identify individuals at high-risk of severe COVID-19 for which shielding is required [43] encouraged the CDG to adopt population health management tools to use data from the SWD to predict rates of Covid-19 vaccine uptake, before Covid-19 vaccines became available, based on historical influenza vaccine uptake data. This allowed the identification of groups and locations where people were more likely to be reluctant to get the Covid-19 vaccine, and subsequently, adapting the vaccination programme to meet the needs of those individuals and communities [26]. The SWD continues to be used to inform the Covid-19 and influenza vaccine programme design and delivery to date.

*“Having that data and intelligence enabled me to say well actually these are the places where people aren’t coming forward to be vaccinated. It’s the only thing I have ever worked on that has had near live data availability between organisations”.*

*“Data was used extensively to reinforce the targeting of (vaccination) activity”*.

*“It (analysis of historical flu vaccination) was the basis for evidence for starting to create clinics in communities”*.

**Fig 1:**
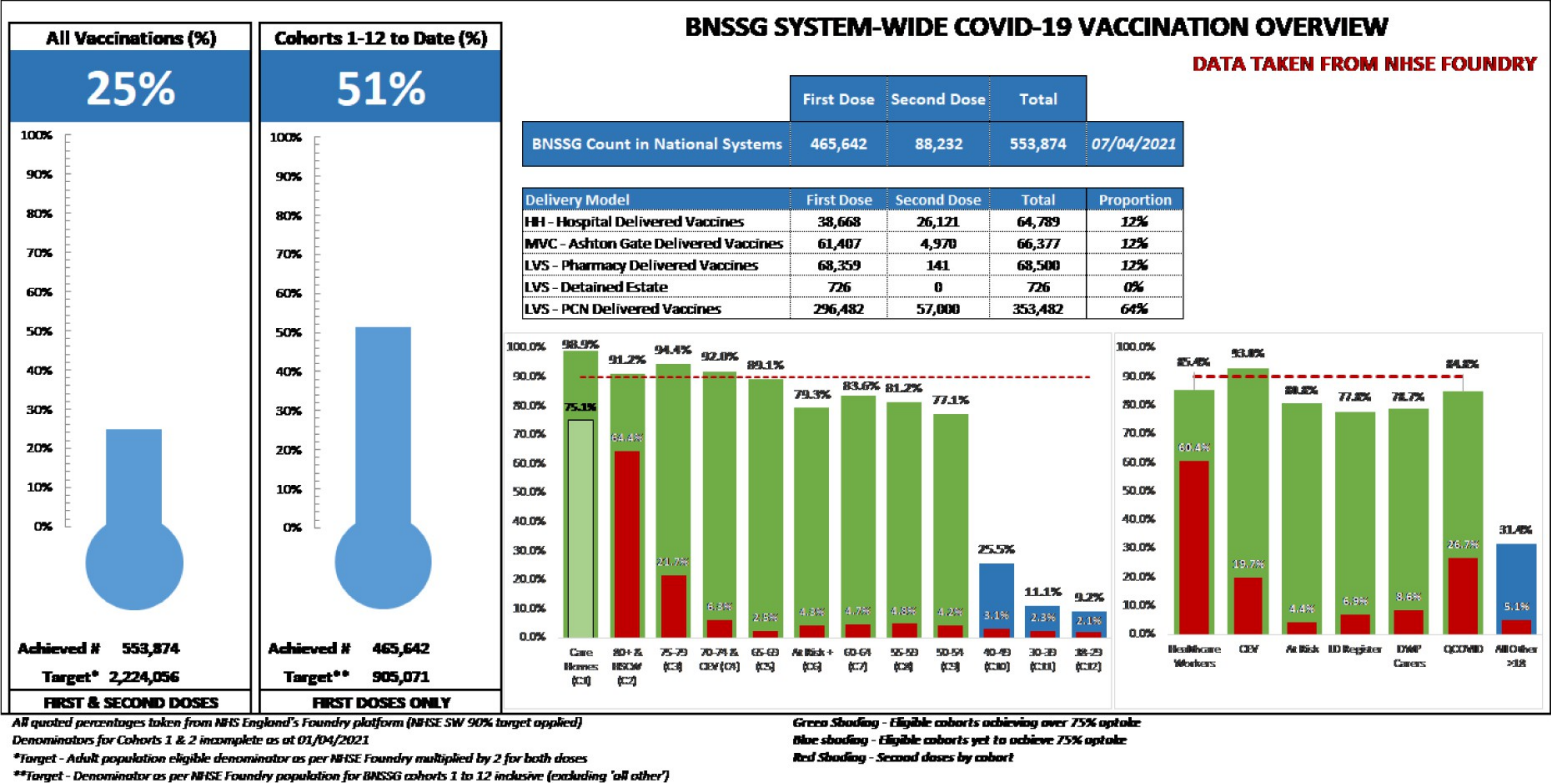
An example of a daily progress report shared in the daily CDG meeting.

**Fig 2:**
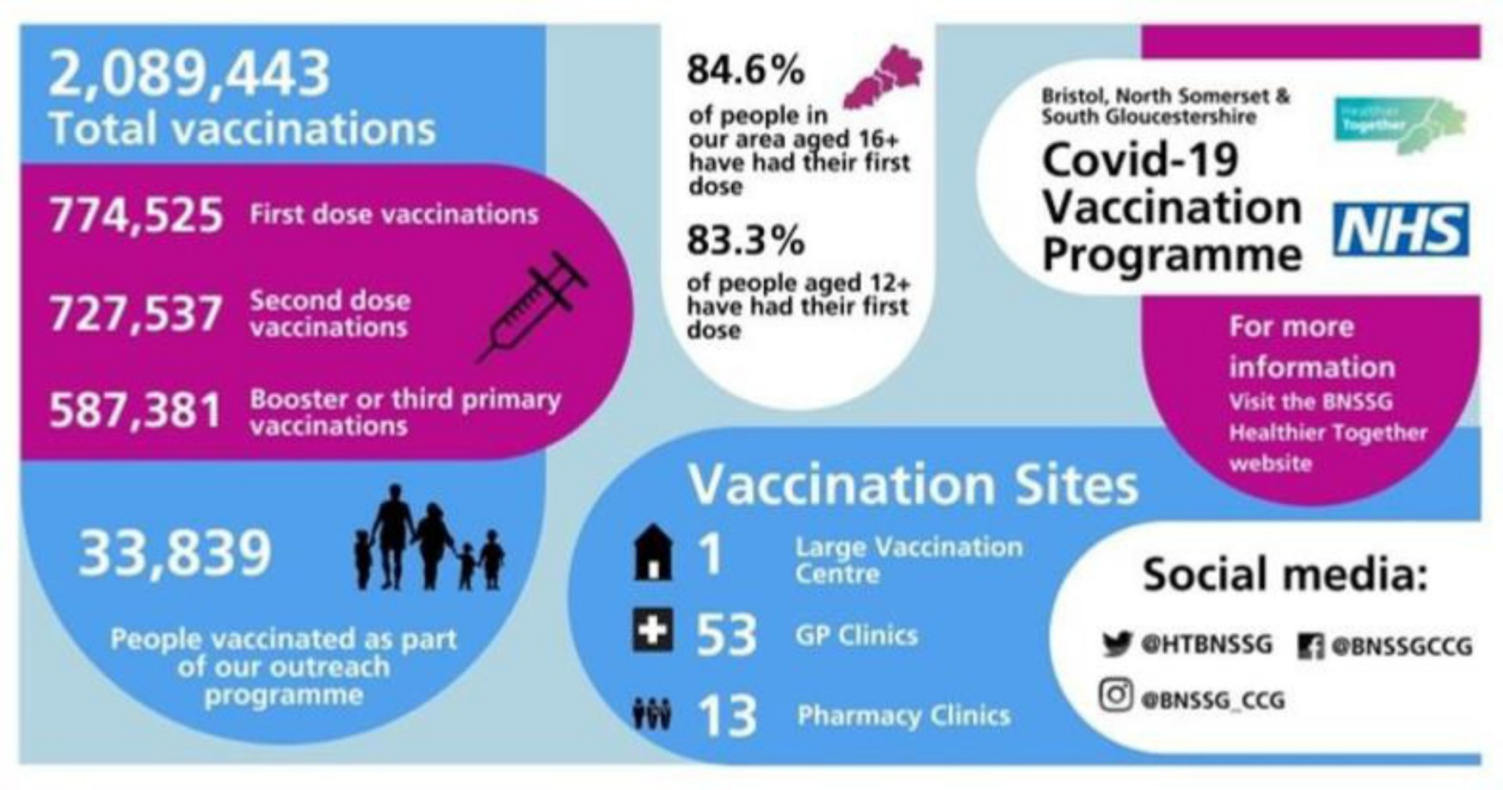
Weekly infographic showing BNSSG vaccination uptake (3^rd^ March 2022).

In addition to using national and local data to tailor vaccination efforts to meet the needs of individuals and communities, sharing vaccine uptake data across the organisations delivering the vaccination programme, and visualising this data in a meaningful way have led to continuous engagement from the organisations and motivation to meet the needs of people and groups lagging behind (see Figs 1 and 2). Noteworthy, prior to Covid-19, there were restrictions on the sharing of data outside of NHS and ICS partners. However, the Secretary of State for Health and Social Care issued Notices under Regulation 3(4) of the Health Service (Control of Patient Information) Regulations 2002 (COPI) requiring organisations to share confidential patient information with organisations entitled to process this data under COPI for COVID-19 purposes (COPI Notices).

*“The rich population health data (and the ability to share across partners) historically for flu vaccinations, has enabled us to pinpoint health inequalities and direct resources at the right groups and wards across BNSSG”.*

*“Getting a nice dashboard was really important for me*. *We started with a thermometer*, *like a charity thermometer*, *showing us how… I think it ended up in various other more sophisticated graphs*. *But just being able to pause and see on any particular day how many vaccines had been delivered (injected)*. *We got to 100*,*000 then 700*,*000 then the million milestone then the 2 million milestone… and you feed on the positivity*. *You are getting fantastic results and a lot of energy from a lot of people”*.

Visualisation of progress, in the format of number of Covid-19 vaccines delivered, was not only important for CDG members and organisations, but also the vaccination team vaccinating people in vaccination clinics and centres. The vaccinating staff associated the number of Covid-19 vaccines delivered to the number of lives saved; one Covid-19 vaccination injected equates to one life potentially saved.

*“This doctor said to me I’ve probably saved more lives doing this vaccine that I have in all my career as a doctor”*

*“It’s quite sad really but in 25 years of working in the NHS the last two years have provided the first link between what I do, day to day, and a visible and tangible direct benefit to patients and the wider population.”*

*“How often do you know how many lives you’ve potentially saved today*?*”*

A key lesson for the future is to harness the potential of digital technologies to facilitate the capture of the data necessary to inform decision making. National data availability does not substitute the need for local context parameters to be captured to inform the implementation effort locally. Furthermore, for big and complex interventions, presenting progress data in simple ways (rather than complicated diagrams) will help all stakeholders, at every level, to make sense of it and use it to drive the implementation forward.

#### 4. A dedicated communications, insights & engagement team

The BNSSG Covid-19 vaccine programme had a dedicated Communications, Insights and Engagement (CIE) team, which was seen by the participants as instrumental to the design and delivery of the Covid-19 vaccination programme in BNSSG. Representatives from this team have been part of the CDG since the early phases of the project. In addition to data from the SWD, the CDG decision making was underpinned by insights gathered by the CIE team through a variety of data sources. These included interviews with patients and members of the public, focus groups with stakeholders, and a large number of pre-and post- vaccination clinic surveys, to add context to the vaccine uptake data obtained from national data and the SWD.

**Fig 3:**
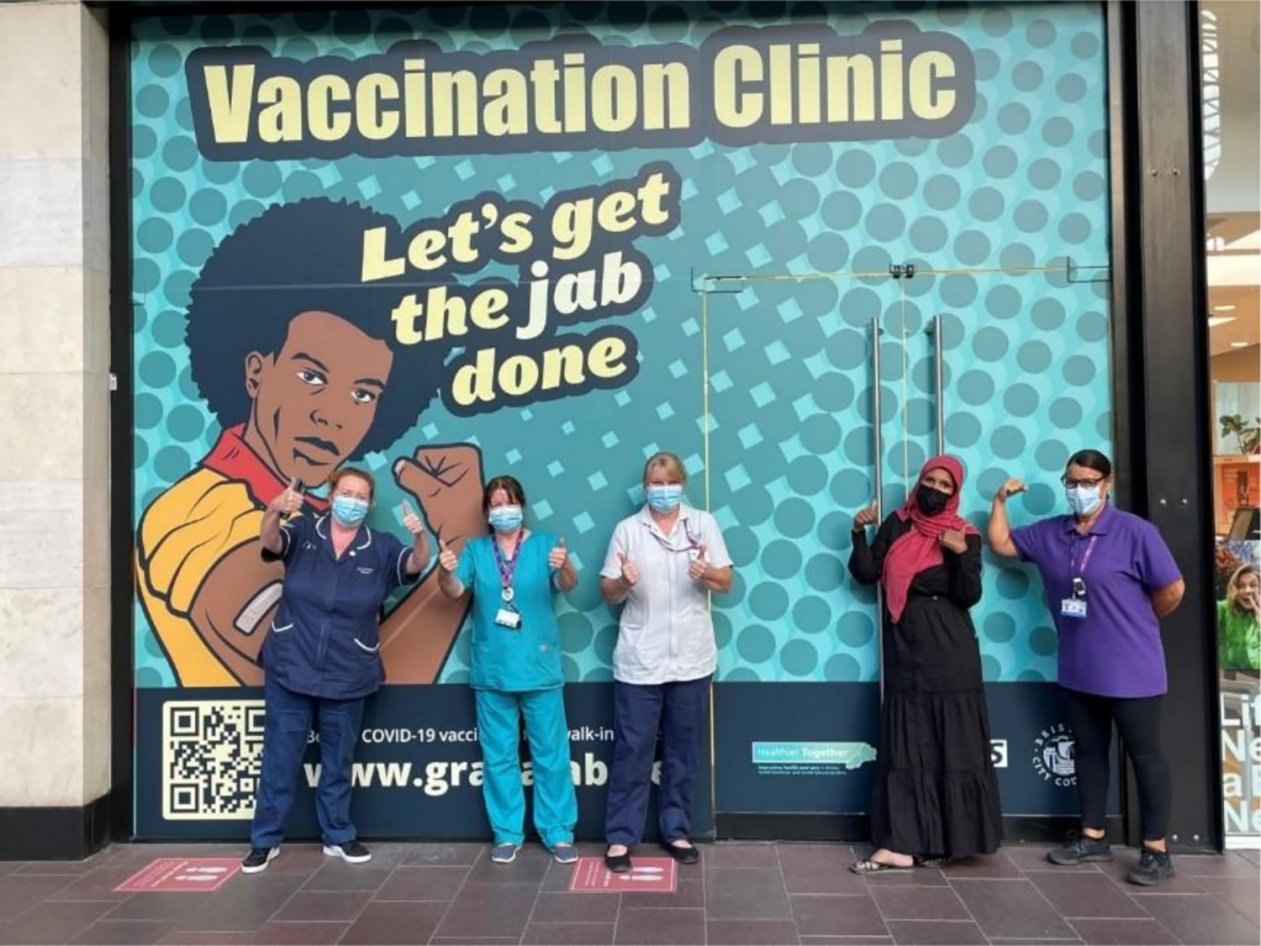
Example poster created by the BNSSG CIE team to encourage Covid-19 vaccine bookings in BNSSG, displayed at the front of a vaccination clinic inside Cabot Circus Shopping Centre in Bristol, UK.

The CIE team worked with the CDG and all the Covid-19 Vaccination Programme partners to refine the public facing message of the programme, created and solidified the programme brand, and focused communication and engagement messages around the programme goal to get as many Covid-19 jabs, in as many arms as possible as quickly as possible. The CIE team ensured the centralisation of communication (www.grabajab.net), and ensured that the vaccination programme appears as one NHS service that can be accessed through multiple providers (See Fig 3 for a poster example). The fact that vaccination commissioning models are different across hospital hubs, primary care networks, mass vaccination sites, pharmacies, children immunisation, and outreach clinics remained behind the scene, for patients and members of the public to see one service. This was further enhanced by a local memorandum of understanding allowing ICS partners to share staff and resources between organisations within BNSSG.

*“They created that sort of unified branding, consciously creating that and it meant that people just saw it as an NHS service provided and the roots of accessing may be different, but it’s all the same. I think that was really, really helpful.”*

*“I think that our local comms and having a dedicated and agile comms team for this project has worked well. Sometimes with comms, it’s difficult because things take a long time to get sorted…but generally it was much quicker than other comms stuff that I have been used to.”*

*“One of the things that is the shining light of this for us is our comms team*. *Because the comms team have always had very much this system lens*. *So*, *they never at any point would look which commissioning model you are a part of*, *they have been really proactive…more looking at the communities we need to reach*, *who do we need to speak with*, *and I think they’ve really helped to knit together the narrative of what we are doing for our people in our local area*, *as a joint effort*. *And I think they’ve been really good*.*”*

The CIE team’s work has also been pivotal to the success of the Maximising Uptake Programme [18]. The team worked closely with science communication specialists, the media, community leaders and members of the public to engage with people and communities reluctant to get the Covid-19 vaccine [18].

*“What a great relationship BBC Points West has built up with (the head of the CIE team). Her ability to work with us is fantastic! Always clear and open to what we need to make great telly with clear messaging!”*

*“People trust the messenger sometimes more than the message…So we identify the right messengers across communities where we know, there is a low uptake and maybe misunderstanding and distrust around the whole COVID and the vaccine. Then we build on that asset, we build on that trust and relationship”*

*“I really get the impression they try to go out through*, *the local community routes*, *right down to WhatsApp’s and multiple languages … using texts and videos and posters and memes and all kinds of things*. *I think that’s been really fantastic*, *to try and get the message out to as many people as possible”*.

The importance of a strong communication and engagement strategy to accompany the implementation of complex interventions could not be over-stated. Harnessing the skills and expertise of communication experts to develop the brand of the intervention and maximise its clarity, reach and impact is a key driver to the successful implementation of complex interventions. This is because it ensures all the stakeholders, at all levels, understand the intervention and remain focused on its goal throughout the implementation journey.

#### 5. Individual skills and expertise

The programme brought together a diverse group of people, with a varied skill set and backgrounds and experiences beyond the healthcare sector. These included people from the aviation industry and manual labour jobs who have been furloughed during the pandemic. The programme leadership empowered individuals at every level to innovate and lead on tasks that could be done better than planned, and enable achieving the programme’s goal faster. Consequently, many people were able to innovate and lead on programme delivery.

*“It has [the programme] shown me what my skill set really is [leadership]”*

*“It’(the programme) s been a game changer for me. Made me feel more confident and given me exposure to high level system issues and conversations”*

*“To start with*, *even the way we were to operate the [mass vaccination] centre was mapped out by national [the national team]*. *The people that designed the map of the mass vaccination centres were military based and trained*. *It had that feel about it… Before we even opened*, *we said hmmm*, *that ain’t kind of gonna work*! *But they [the national team] were really strict and we had to toe the line*, *we had to go with it*. *Then of course*, *within a day or two*, *we worked out that we could do it in a better way…That patient flow*, *patient pathway*, *changed quite dramatically from what was the dictat right at the start… With support*, *we could say no*, *we are going to do it in a different way*, *and it will be ok*.*”*

The leadership skills of many people on the programme enabled them to identify the set of skills and expertise required to deliver programme tasks, and actively engage with people to harness their diverse backgrounds and expertise.

*“We had really experienced staff who had come from all different backgrounds and walks of life, and they had lots of different experience in different things. Some of them were really skilled, like St John’s Ambulance (a charity organisation). They could do vaccinations because they had done the training. Even non-NHS people would suggest things; can we try this or that? We’d say let’s try it. And if it worked, great. So, it was all about working with what you had using the people that you had and their skills, to try and change things for the better.”*

For example, as part of designing patients’ flow, the security team at mass vaccination centres were consulted on the best way to manage this as they had expertise in this area. A member of the security team said:

*“You don’t feel they are talking down to you, you feel like they are asking your opinion, your view. It was great for them to ask for us, and to keep being a part of it.”*

There were many talented individuals within the programme with skillsets that were critical when managing crisis meetings and exploring solutions to difficult and unprecedented situations. Managing the communication process itself in crisis management meetings is essential to enable collaboration and problem solving, and this was noted by many participants.

*“The chair has been a fantastic facilitator of the whole process. At times, the process has felt challenging and confusing, but she has made it run smoothly.”*

*“The Q&A (question and answer) sessions were and still are amazing. They brought everyone together.”*

*“I think sharing good practice across BNSSG at our meeting chaired by… worked well*. *If there’s a problem we solve it together as a group*, *that’s worked well*.*”*.

For complex interventions to be successfully implemented, a balance between central command and control and wider empowerment has to be achieved. Big organisations such as the NHS employ many people with wide ranging backgrounds, skills and expertise. With appropriate governance, complex interventions have to have “built-in” mechanisms to empower people at all levels to use their skills and expertise to adapt the programmes and their implementations to their settings.

#### 6. Financial resources

The funding of the BNSSG vaccination programme has undoubtedly been a key driver to the success of the programme. Prior to Covid-19, commissioning services were time consuming due to the cumbersome bureaucracy associated with requests for funds. However, the national Covid-19 programme ensured the availability of equipment and logistical resources to set up vaccination clinics quickly. Furthermore, the process of requesting funds to support vaccination related activities was simplified and funding decisions were made quickly. These funds were used to support communication and engagement campaigns and fund clinicians e.g. general practitioners and other stakeholders to attend programme meetings and input into a unified vaccine delivery model.

*“A main benefit of the vaccination programme was that it had money attached to it, so then everybody said: “how can I help? Not why I can’t help”. For me, that is the biggest part of the success story. It’s the whole system working with the brief of how can I input into this task and it’s not going to cost me because the money is there. So, how can my bit of the system help?”*

The lack of budgetary constraints meant that the programme could dedicate more financial and other resources to the Maximising Uptake part of the programme [26], to enhance the uptake of Covid-19 vaccine among underserved communities.

Noteworthy, managing resources and funds in the context of a public health emergency is a key attribute of the programme leaders who have allocated these to tackling health inequalities, an area that has not traditionally been a priority for funding prior to Covid-19. Many programme leaders argued for the need to reframe the costings concept to also account for the risks associated with lack of engagement from marginalised and underserved communities in relation to hospital admissions, deaths from Covid-19, and wider health outcomes.

*“you can’t sort out inequality with a small and non-recurring budget. It’s not going to work. The success [of the programme] has been due to a layering of many resources, but significant funding has been a big part of it.”*

The implementation of complex interventions requires adequate financial resources and a degree of autonomy for local teams to decide on the activities to commission and fund to facilitate the implementation.

### Moving forward

The participants agreed on the unprecedented scale of the BNSSG vaccination programme and their overwhelmingly positive involvement in the programme. However, many are concerned that partnership and collaboration among healthcare and non-health care organisations to deliver tangible outcomes to people in the region will be lost now that the sense of Covid-19 urgency is subsiding.

“*It (the programme) has helped me imagine a way of multi-agency working that returns to first principles of what is possible, rather than working in silos, harbouring suspicion and fear of excessive workload*”

“*I think there is a tension when the whole nation is in a tight spot*. *And delivering care in that context was very intensive*. *Now*, *it’s very easy to go back to familiarity*. *So just now we need to encourage and find those new ways of working*. *We need to almost have an expectation that this is how people should work together*.”

Empowering people at all levels to suggest solutions, innovate and lead on programmes and initiatives has also been suggested by the participants as a key lesson to apply to future programmes and interventions.

*“For too long the NHS has been too top down in its approach. This programme has really shone a light on how successful working from the bottom up can be. And how it can really change communities. Going forward, that should be a key learning that is taken forward to address other health agendas”.*

*“At the very base of any future change is something about culture empowerment*. *Empowering and valuing your workforce*. *But as I am saying it*, *I am rolling my eyes internally*. *It’s leadership 101*. *It’s part of the NHS Leadership programme*. *The NHS knows it*, *it just doesn’t do it*.*”*

## Discussion

This study explored the perspectives and experiences of a large number of stakeholders involved in the design and delivery of the Covid-19 vaccination programme in Bristol, North Somerset and South Gloucestershire in the Southwest of England, UK. It aims to highlight the key drivers, from stakeholders’ perspectives, that contributed to the success of the Covid-19 vaccination programme, which has led to more than 2,650,481 Covid-19 vaccinations given. Insights from the literature on how different organisations/ teams in the UK and internationally have implemented Covid-19 vaccination programmes are limited. Therefore, our study findings are important in order to highlight the lessons learnt from this endeavour that could inform interventions in future emergencies.

To our knowledge, this is the first study to investigate the implementation of a large Covid-19 vaccination programme from a multi-level and multi-professional perspective, using the NPT to explain the drivers to the successful implementation of the programme. A further strength of this study is the relatively large sample size (n = 75) and the representation of multiple stakeholders within this sample [44]. The findings of this study may be transferrable to similar healthcare contexts internationally, however, we make no claim regarding their generalisability. Self-selection bias could influence the findings of the study based on the characteristics and the topics put forward by those who chose to participate in the study [45].

This discussion focuses on the drivers to the successful implementation of the Covid-19 vaccination programme in BNSSG. A discussion of the NPT domains, the programme capability and context is presented in S1 Appendix.

### A leadership team

Teamwork within the Clinical Delivery Group was critical to the successful implementation of the programme. The group consisted of members from organisations involved in the implementation of the programme, with complementary skills and expertise; who were committed to vaccinating every eligible person within BNSSG against Covid-19, were concerned with vaccine uptake rates; and held themselves (individually and as a group) accountable to the approaches used to implement the programme. This aligns with Katzenbach and Smith’s definition of high performing teams [46]. In their Wisdom of Teams book, which was based on data collected on 50 different teams in 30 companies, they highlight the importance of a meaningful purpose for teams to perform well [46]. The purpose of the CDG team was shaped by the Covid-19 public health crisis, and the notion that vaccination offers protection against the disease and a way back to life before the pandemic; out of lockdowns, social distancing and travel restrictions [47]. The purpose to vaccinate as many people as possible against Covid-19 was translated into a specific performance goal, that was measurable and trackable throughout the lifetime of the programme and the team. This is argued by Katzenbach and Smith as a key feature of high performing teams, that are able to align their purpose with their performance goals [46].

The CDG provided a safe space to share ideas and concerns which ensured psychological safety; “the extent to which team members perceive that they can take interpersonal risks such as speaking up, admitting a mistake, acknowledging confusion and offering a dissenting opinion without undue risk of being punished or rejected” [48]. This psychological safety is critical to facilitating creativity and enhancing effectiveness and performance [49]. It can be achieved through creating “challenging but not threatening” spaces where “blame is replaced with curiosity”. This in turn creates trust and fertile grounds for innovation and ground-breaking solutions [50]. The CDG granted flexibility to vaccination teams, under a vigilant clinical governance, to design and deliver their vaccination clinics in the way they see fit to deliver on the programme goal. They also organised regular meetings and events to present updates on vaccination efforts across all the local vaccine delivery sites and share learning and good practice. These meetings have been shown in other contexts as enhancers of teams’ performance [48]. Furthermore, this information sharing among all stakeholders, being heard and understood as equal partners, and taking part in shared decision-making are suggested by Norris et al. (2017) as key ways to ensure active engagement among stakeholders from multiple hierarchical levels within complex healthcare systems [51].

### Communication, insights and data

The clarity of purpose and its alignment with the programme performance goals have been achieved through harnessing the skills and expertise of communication experts to develop the brand of the programme and maximise its clarity, reach and impact. This ensured that all the stakeholders, at all levels, understood and remained focused on the purpose of the programme throughout its implementation journey [52]. This specificity of the programme’s goal or purpose facilitated clear communication and focused discussions on the efforts or interventions required to achieve this goal [46]. It also meant that stakeholders and vaccination staff on the ground remained compelled, motivated and energised to increase the number of people vaccinated against Covid-19. Tannenbaum et al. highlights the importance of communicating wins and successes to help stakeholders believe that success is tenable, boost morale and a sense of achievement, and to sustain programme efficacy [53]. Progress reports that are simple, clear, without jargon, or complicated visuals or the need for further explanation are a key tool in a crisis context. Data visualisation in public health is key during outbreaks to facilitate the interpretation of data and direct response [54]. In a fast-moving, potentially rapidly changing intervention in response to a public health emergency, it is vital that all stakeholders are able to quickly and easily access, process and understand key information in order to implement resulting required actions in practice on short timescales. While these take time to produce, they can be updated rapidly with daily data once the format is created, and positive effects on the workforce during a crisis have clear value. Communication expertise are also needed to collaborate with local people and communities to understand the needs and concerns of individuals and groups that have a low confidence in Covid-19 and other vaccines, and tailor vaccination messages to address them [26, 55].

Surveillance and other data to predict outbreaks, vaccination rates and outcomes have played an important part in the successful implementation of many Covid-19 and other vaccination programmes around the world [47]. Local insights and routinely collected datasets can inform the adaptation of national programmes and initiatives to local contexts [26, 43, 56, 57]. Furthermore, the availability of local data and insights allows quick decision-making to direct local implementation when the availability of national vaccination rates data lags. Parameters such as ethnicity have historically been poorly captured in health and social care data in England [58]. This has often led to an incomplete or inaccurate estimation of potential groups that are likely to be severely impacted by Covid-19, and in need of vaccination prioritisation [58]. It also highlights the need for local data and surveillance to be enhanced to enable efficient planning in future emergencies.

### Partnership and empowerment going forward

There was a clear sense among our participants that it is desirable for both the collaboration among healthcare and non-health care organisations to deliver tangible outcomes, and empowerment of people at all levels to contribute ideas and take on leadership, developed in response to the urgency of the Covid-19 situation to continue into everyday future working. Multi-organisation and multi-sectoral collaborations are pre-requisites for an efficient and timely responses in public emergencies [59–61]. Furthermore, collaboration with organisations from the Voluntary, Community and Social Enterprise (VCSE) sector have proven invaluable during the Covid-19 pandemic where their expertise, networks and resources have been harnesses to design, deliver and evaluate vaccination clinics and health education and promotion campaigns [62]. Place-based partnerships and strong relationships between health and social care organisations and local authorities have been hailed as significant success stories of the roll out of the national Covid-19 vaccination programme, with strong calls to preserve these relationships and partnership working moving forward [63].

In this study, two key culture shifts that would enable positive changes to propagate forward from the experiences of rapid adaptation and implementation in the Covid-19 pandemic were evident. First, an expectation that people will work together, with or without the intensity of a national crisis would enable people with a range of backgrounds to collaborate effectively and efficiently towards common goals as standard practice. This, in contrast to the reported “familiarity” of working separately or with apprehension about partnership working, would allow optimisation of knowledge, skills, processes time and resources within and between organisations [63, 64]. This is further underpinned by the need for continued empowerment at and valuing of all levels of the workforce [65, 66].

Through a shift towards collaborative, partnership working and ‘flat hierarchies’ being seen as normal and desirable working practices, the resulting flexibility, optimisation and motivation reported in this intervention could thereby translate into various other contexts in which decision-making and implementation, including across complex situations, are needed. Within this, the funding of the BNSSG vaccination programme was undoubtedly a key driver to the success of the programme, underpinning the layering of multiple differing resources. A need for access to continued, long-term funds, rather than a “small and non-recurring budget” to address inequality was highlighted in comparison to the positive outcomes of this programme with its simplified, available and responsive access to funds. The momentum generated by the national and local Covid-19 vaccination programmes is palpable. NHS England and NHS Improvement are now providing the Health Equalities Partnership (HEP) Programme funding to local systems to tackle health inequalities [67]. Within BNSSG, various small funding schemes have been launched to support community organisations to tackle health inequalities. Although these resources remain limited, they are a first step in direction of empowering people and communities to address their health inequalities.

## Conclusions

This study highlights a number of mechanisms that can be deployed in future crises to implement large, complex interventions on the ground. Below are the key points arising from this evaluation:

- The aim or mission of the complex intervention should be articulated in simple words that do not contain any jargon. It should stand without further explanations or objectives, and should remain consistent throughout the implementation of the intervention.
- The skills and expertise of communication experts are key to developing the brand of the intervention and maximising its clarity, reach and impact.
- The potential of digital technologies should be harnessed to facilitate the capture of the data necessary to inform decision making.
- National data availability does not negate the need for local context parameters to be captured to inform the implementation effort locally.
- For large and complex interventions, presenting progress data in simple ways (rather than complicated diagrams) will help all stakeholders, at every level, to make sense of it and use it to drive the implementation forward.
- Visualisations of progress, as well as being informative for leadership teams, can foster a sense of positivity and achievement for those implementing interventions ‘on the ground’.
- Collective leadership, through a multi-stakeholder team with representation from all organisations involved in the implementation of the complex intervention ensures that these organisations are part of the implementation decision-making process. This can also strengthen their commitment to implementing the intervention, and provides a mechanism through which they can appraise and adapt the intervention to ensure its successful implementation.
- For complex interventions to be successfully implemented, a balance between central command and control and wider empowerment must be achieved.
- Operational flexibility is key to responding effectively to local variations and needs.
- The implementation of complex interventions requires adequate financial resources and a degree of autonomy for local teams to decide on the activities to commission and fund to facilitate the implementation.

## Supporting information

S1 Appendix The mapping of themes emerging from the qualitative data to the domains of Normalisation Process Theory is presented in S1 Appendix. (DOCX)
