# Supplementary material for: Lessons learnt from the implementation of the Covid-19 vaccination programme in the Southwest of England
Source: PLoS One. 2024 Aug 28;19(8):e0309230. doi: 10.1371/journal.pone.0309230 (PMC11356399; doi:10.1371/journal.pone.0309230)
Supplement: S1 Appendix — (DOCX) [file pone.0309230.s001.docx]

**Appendix 1**

**Table 1. Mapping of themes to NPT domains**

| **Theme** | **Definition** | **NPT Domains** |
| --- | --- | --- |
| One clear, measurable goal | Clarity and simplicity of the programme goal: Vaccinating as many people as possible as quickly as possible. | **Context:** Vaccination was the only way out of the Covid-19 crisis.  **Capability:** Vaccination programmes as part of routine clinical practice  **Coherence:** Havin one clear, simply articulated goal helped people understand and articulate the intervention. |
| One multi-disciplinary leadership team | Setting up a multi-professional team to oversee the programme design, implementation and adapting it to the fluid Covid-19 crisis. The team provided:   - Leadership; - A safe space to share concerns and be creative; - Mechanisms to adapt the programme to the local narrative; and - Governance to allow operational flexibility when implementing the programme on the ground. | **Context:** The implementation of the programme requires co-ordination from multiple stakeholders locally. Their representation in the group overseeing decision making is key to facilitating the programme implementation locally.  **Coherence:** All stakeholders understood the programme and its goals in the same way. All parties were working towards the same purpose.  **Cognitive participation:** Multi-professional, and multi-stakeholders’ representation within the leadership group reflected and ensured engagement and commitment of organisations to dedicate time, energy and resources to facilitate Covid-19 vaccination clinics and monitoring of vaccine uptake. This team met daily.  **Collective action:** Th group provided collective leadership of the programme. Members of the group provided insights on their respective organisations’ actions, the impact of the programme on their routine practice and solutions to troubleshoot arising implementation challenges.  **Reflexive monitoring:** The team met daily to discuss feedback from teams implementing the programme and action any issues highlighted. The team also monitored vaccine uptake and adapted the implementation of the programme (by redirecting resources) to areas/ groups where vaccine uptake was (or was predicted to be) low. |
| Data-informed decision making | The use of a local data to review historic influenza vaccination uptake rates and predict geographical areas and patient groups likely to be reluctant to accept the Covid-19 vaccine. This then informed decision making on the design of outreach activities and bespoke clinics, and allowed adaptation of the vaccination programme to the local context. | **Context:** The use of the local data alongside national data “articulated” the local context and allowed the adaptation of the Covid-19 vaccination programme to address local low uptake rates of the vaccine in BNSSG and tailor efforts to maximise the reach and acceptance of the vaccine.  **Capability:** The local dataset was launched in August 2019, prior to the first case of Covid-19 being confirmed in the UK on 29^th^ January 2020. The dataset was developed in order to link together information across primary care, secondary care, mental health and community services for over 1 million patients in BNSSG. Therefore, the intention to use an integrated dataset to inform local decision making and commission was established, and intended to be routine practice, not long before the start of the pandemic. Soon after, the dataset was used to predict the risk of severe Covid-19 and the need for shielding. This then allowed the use of the dataset and other local data to adapt local interventions and routinise them.  **Coherence:** The use of data to track Covid-19 vaccine uptake locally, and visualising it in a meaningful way, further re-enforced the aim and the articulation of the intervention. All stakeholders understood that the programme is about vaccinating as many people as possible, as quickly as possible. All organisations were working towards the same purpose, which was strongly and clearly represented by the data.  **Cognitive participation:** As the data continued to demonstrate Covid-19 vaccine uptake patterns, organisations participating in the implementation of the vaccination programme remained engaged to address low vaccine uptake rates and propose solutions and resources to maximise it.  **Collective action:** The organisations implementing the vaccination programme individually and collectively strived to address low vaccine uptake rates, including sharing resources (such as vaccine supply) amongst them to enable the whole group to reach the programme aim.  **Reflexive monitoring:** The use of the data allowed tracking vaccine uptake rates and provided instant feedback on the success/ failure of the programme. Reports on implementation progress from the participating organisations were discussed daily and issues identified were addressed promptly. These reports included feedback from the ground and all layers of the organisations. Actions were fed back all the way down to the ground (at clinic level). The data allowed the standardisation of communication around a common theme, Covid-19 vaccine uptake rates, and how to maximise them. |
| A dedicated Communications, Insights & Engagement (CIE) team | The CIE team augmented the data from the local dataset with data collected through focus groups, interviews and pre-and post-vaccination clinic surveys. The team also created a unified “brand” for the vaccination programme and collaborated with local communities to co-create culturally competent myth-busting and vaccine information resources. | The data contribution of the CIE team is included in the mapping of data-informed decision making against the NPT domains.  **Context:** The Covid-19 pandemic has been dubbed as a pandemic of misinformation [1]. Unlike previous pandemics, technology has made it possible for (mis) information to be shared in a variety of formats and through readily accessible social media platforms. Throughout the pandemic, while healthcare professionals and governments were trying to navigate the uncertainty of the condition and the literature on Covid-19 effects, the vaccines, their effects and side effects, people all over the globe were communicating and sharing their experiences, fears, concerns, misconceptions and opinions in a way that continues to affect people’s intention to get vaccinate to date. The realisation that information and communication about Covid-19 and its vaccines are as important as the vaccines themselves led to the inclusion of a dedicated communication team in the design and implementation of the BNSSG Covid-19 vaccination programme.  Another element of context of the BNSSG Covid-19 vaccination programme relates to the prevalence and impact of health inequalities on trust in, and engagement with, the Covid-19 vaccination programme [2]. Typical models of communicating healthcare information, where the healthcare organisation creates and communicates information to people in the main language or translated word for word to other languages, did not succeed in convincing underserved communities living in deprived areas on the safety of the vaccines; easier to access information (appropriate or inappropriate), from ordinary, relatable and influencing people was more convincing. The CIE and the whole BNSSG Covid-19 vaccination leadership team needed to think of novel ways to reach communities and fight against the tide of misinformation stopping people from getting vaccinated. This context element was encountered during the programme implementation and as a result, programme delivery had to be adapted to address this challenge [2].  **Capability:** The CIE unit had been part of the BNSSG Clinical Commissioning Group (now called an Integrated Care System*) since its establishment in 2013. However, many Communication and Engagement teams in ICSs around the country do not have an Insights expertise within them unlike the BNSSG CIE team**. Although the unit existed before the Covid-19 pandemic, a dedicated CIE team was formed to support the Clinical Delivery Group of the BNSSG Covid-19 vaccination programme. Its main objectives were: To encourage the uptake of Covid-19 vaccines among BNSSG’s diverse population by providing clear, consistent, timely and accessible information tailored to a range of audiences; Design and implement insight-led communications and engagement plans that meet the needs of the local BNSSG communities; Listen to what local communities need and offer the right information, in the right format through the best available channels; Provide insights to inform operation decisions; and understand the impact of the vaccination work through evaluation and feedback from patients, partners and communities. This meant that the CIE team’s input in the BNSSG Vaccination Programme was aligned with their routine practice.  **Coherence:** The CIE team was instrumental to formulating the branding of the vaccination programme and facilitating its understanding and articulation. The team was also able to rapidly adapt national Covid-19 vaccination messaging so that it accurately reflected the local programme and considered nuances and concerns raised by BNSSG population as vaccination was rolled out.  **Collective action:** Developing a unified branding of the programme and streamlining the programme information and booking systems onto one single front meant less need for individual organisations to develop their own communications (i.e. minimised the impact of the adapted programme on their routine practice), a streamlined collective vaccination effort, and that people to be vaccinated are receiving the same information, irrespective of where they live within BNSSG.  **Reflexive monitoring:** The CEI team provided many of the feedback and evaluation mechanisms to appraise the programme. This was done through extensive engagement with people and organisations and exploring their experiences of setting up and delivering vaccination clinics and getting vaccinated in those clinics. |
| Individual skills & expertise | The Covid-19 Vaccination Programme required the recruitment of a large workforce of vaccinators and support staff. The BNSSG Covid-19 vaccination programme leadership team provided the governance and mechanisms to allow individuals at all levels of the programme to co-design and implement the programme. Individual skills, past experiences and expertise were sought and empowered to improve processes within the programme to enable its effective delivery. | **Coherence:** People with varying clinical and non-clinical backgrounds, skills and expertise understood the purpose of the vaccination programme in the same way and tailored their input to allow an effective design and implementation of the vaccination programme.  **Cognitive participation:** Empowering staff to be actively involved in the programme’s design and implementation ensured that their contribution to the programme evolved and was being tailored to respond to the challenges and new issues arising throughout the programme.  **Collective action:** Individuals and groups’ efforts to implement the vaccination programme were underpinned by their empowerment to use their individual skill sets to solve problems encountered when designing or implementing vaccination clinics, with overall governance from the programme leadership team.  **Reflexive monitoring:** People were encouraged to appraise and/ or challenge directives and decisions that did not work on the ground, and adapt the implementation as needed on the ground to maximise efficiency and vaccine delivery. |
| Financial resources | It is currently estimated that the Covid-19 national vaccination programme costs mount up to £11.7 billion. Decision-making relating to the management of financial resources within the programme was a key driver of the success of the local vaccination programme. Programme leaders re-defined the value for money concept and dedicated financial resources to co-design culturally competent communication resources with the local communities and develop bespoke interventions to tackle low uptake rates due to health inequalities. | **Context:** The national Covid-19 Vaccination Programme was unprecedented in terms of scale and financial support which consequently meant that the local programme was well resourced. People experiencing (or at risk of) health inequalities are most at risk of dying or having a severe infection. They also tend to be less likely to engage with vaccination efforts [3]. Directing financial resources to facilitating access to and acceptance of vaccination is important to reduce hospital admissions, intensive care unit bed use and morbidity and mortality.  **Capability:** Funding programmes and services is routine practice. However, funds are usually small and require a cumbersome application process to se  cure them. Applying for grants to deliver services as part of the Covid-19 Vaccination Programme was less cumbersome and the funds were released quickly.  **Collective action:** Financial support for individuals and groups’ actions throughout the Vaccination Programme ensured that the implementation of the vaccination programme aligned with, and did not infringe, on other tasks due to the availability of staff and resource. Funding dedicated to tackle health inequalities allowed the organisations participating in the implementation of the programme, individually and collectively, to dedicate efforts to reduce the impact of health inequalities on people’s access to and engagement with the vaccination programme.  **Reflexive monitoring:** To tackle the impact of health inequalities on access to and engagement with the Covid-19 vaccination programme, the leadership team directed substantial funding to support the design and delivery of bespoke vaccination clinics and outreach activities to increase the uptake of Covid-19 vaccine amongst marginalised and underserved communities. Although these clinics and activities cost significantly more than vaccinating in mass vaccination centres, GP practices and community pharmacies, the leadership team accepted that there are many barriers preventing some communities from accessing these more cost-effective services. Furthermore, the team decided that the high costs of these clinics were still considerably lower than the costs associated with hospital admissions and intensive care unit bed use, prolonged morbidity and mortality among the unvaccinated. This allowed reflexive monitoring of programme delivery, and directing resources as needed to adapt the programme and its implementation. |

*Integrated Care System (ICS): An NHS organisation responsible for planning, in collaboration with other organisations in the locality, healthcare services that meet the needs of the local population and managing the budget for these services [4].

****** The Integrated Care Board of the ICS has now removed “Insight” and “Engagement” functions from the communication team. These functions retain links with the Communication Team, but have been moved to the Strategy Directorate of the ICS.

**Discussion**

**The capability (the workability) of the BNSSG Covid-19 vaccination programme**

Applying the NPT lens, the capability of the BNSSG Covid-19 vaccination programme to be integrated into routine practice stems from its core mission to vaccinate as many people (adults and later on children) as quickly as possible against Covid-19. Vaccination programmes were being implemented in the UK long before the birth of the National Health System (NHS). In 1840, the Vaccination Act was introduced in the UK to provide free vaccination (through variolation) against small pox. Subsequent, sometimes controversial, acts further facilitated the implementation of vaccination programmes [5]. Upon its foundation in 1948, the NHS became the first universal health care system in the world, and in 1958, it delivered the first mass vaccination programme, vaccinating children under the age of 15 against polio and diphtheria, paving the way for many subsequent mass vaccination programmes [6]. Healthcare professionals, patients and other stakeholders are generally familiar with vaccination programmes and how these are accessed through the NHS. Immunisation is offered from 8 weeks of age, and throughout people’s lives especially if they are at an increased risk of morbidity and mortality [7].

Prior to the Covid-19 pandemic, primary care was the main provider of national childhood (pre-school) and influenza immunisation [8]. This means that BNSSG Covid-19 vaccination programme is inherently capable (workable) to be integrated into routine practice since vaccination is part of that routine. However, the pandemic has forced commissioners to extend the provision of vaccination programmes into hospital hubs, pharmacies, mass vaccination centres, and various outreach sites including supermarkets, shops, parks, mosques, churches, hotels, boats etc… [2; 9]. This expansion meant that the “workability” of the programme could be challenged as vaccinating people in these venues, with all the necessary logistics (and the necessary inter- and intra-relationships between the various stakeholders) to implement the intervention in these settings, has never been done before (on such a scale).

**The context of implementing the BNSSG Covid-19 vaccination programme**

Context here refers to the systems that form the dynamic environment in which the intervention is implemented form the context of the intervention [10]. For the BNSSG Covid-19 vaccination programme there are local and national context considerations which often, but not always, align. These contexts relate to the available resources (human, material, and organisational resources), task allocations, social roles, and team norms [10].

It is important to acknowledge that the national Covid-19 vaccination programme (which the BNSSG programme is part of) is unprecedented, as it is the biggest vaccination programme in the UK’s history [4]. Therefore, Hawe et al. (2009) argue that such programmes “may then be seen as a critical event in the history of a system, leading to the evolution of new structures of interaction and new shared meanings” [11]. This adds complexity, and difficulty in ascertaining all dimensions of the context of this programme, not least because of the timing of implementation, in the midst of a global public health crisis. For example, primary care in the UK (including general practice, pharmacies, maternity services, occupational and school vaccinations) has always delivered the bulk of national vaccinations such as (pre-school) childhood and influenza vaccinations. Therefore, they had the required infrastructure and resources to implement the Covid-19 vaccination programme. However, vaccinating people against Covid-19 required bigger spaces (to allow for social distancing), enhanced infection prevention and control procedures, and because of Personal Protective Equipment (PPE) changing requirements and new data capture protocols, the procedure was likely to take longer. This meant that there was need to consider more and larger venues and bespoke vaccination clinics to help increase vaccine uptake quickly [12]. Furthermore, the need to provide these extra clinics required more staff to deliver them, which meant training new staff and using resources from charities (e.g. St John’s Ambulance and the British Red Cross) and voluntary organisations [4]. This, in turn, meant that new relationships had to be forged, to integrate non-NHS staff into NHS routine, and learn how to work together (including defining roles and task allocations; and challenging team norms) to vaccinate as many people as possible, as quickly as possible.

The context, locally, adds an additional layer of complexity in that vaccine uptake (childhood immunisation, influenza and later the Covid-19 vaccine) differs across the UK, for various reasons, including the sociodemographic characteristics of the local population (especially the proportion of minority ethnic populations) and local levels of deprivation [13]. Therefore, local Covid-19 vaccination programmes have to consider these local variations and adapt the implementation of the national Covid-19 vaccination programme to meet the needs of the local populations, using the local resources and knowledge. It is important to note that implementing programmes such as the BNSSG Covid-19 vaccination programme, during a major public health crisis, requires intuitive decision-making [14]. This is defined as decision making based on knowing how to judge situations and make decisions based on prior experiences, AND sensing or having “gut feelings” about situations and issues that may arise [15]. It is argued that to engage in intuitive decision making, local (proximate) contextual information is immensely important to enable decision making [16]. The national programme provides a national oversight of the implementation and supports local decision making through trends’ data, bench marking implementation progress, and developing national summaries of processes and outcomes that can help further develop local initiatives.

**Coherence: Do stakeholders understand the programme in the same way?**

This domain refers to how stakeholders make sense of the programme, its purpose and what is required of them. The theme of *One clear, measurable goal* strongly illustrates the coherence of the programme, and reflects the clarity of the programme and its purpose to all those involved and/ or affected by it. The *Communication, Insights & Engagement team* helped clarify the goal of the programme and ensured that stakeholders maintained that understanding even when the programme was evolving with the Covid-19 pandemic. Coherence was further ensured through establishing *“One multi-disciplinary team”,* with representation from all the organisations involved in its implementation, who provided (collective) “*leadership”* of the programme. This ensured consistency in how stakeholders perceived the problem that is being addressed by the programme and what was required of them to successfully implement it. This was further enhanced by the use of local data and adapting the programme to the local context as explained under the “*Adapting the programme to the local narrative*” and “*Data-informed decision making*”.

**Cognitive participation: Are stakeholders committed to implementing the vaccination programme?**

This domain refers to the efforts that stakeholders dedicate to continually engage with and support the implementation of the programme. This was facilitated by representation from all stakeholders in the multi-disciplinary team that provided leadership of the programme. Since the stakeholders were part of the decision-making process, this ensured their continued commitment to successfully implement the programme. This is further explained under the “*One multi-disciplinary team”* and *“leadership”* subtheme. Furthermore, the use of local data to inform decision making, as explained under the *“data-informed decision making”* theme, enabled the stakeholders to review their performance (or contribution to the implementation of the programme) and this ensured that they were able to identify and dedicate the efforts required from their organisations to enhance vaccine uptake rates. The cognitive participation in the implementation programme was also ensured through empowering people at all levels to utilise their *skills and expertise* to implement the programme, strengthening their sense of ownership of the programme and leadership on its implementation.

**Collective action: What did individuals and groups do to implement the vaccination programme?**

This domain refers to individual and group actions to implement the vaccination programme. Almost all the themes described below illustrate the actions individuals and groups undertook to implement the programme. Stakeholders were part of the implementation decision making processes and were provided with the context and resources required to contribute to the implementation of the programme. These resources include: skills and expertise, support with communication, appropriate funding, local and national data, space for implementation issues to be discussed honestly and safely, freedom to innovate, guidance and governance from a multi-disciplinary team representing all the stakeholders involved in the implementation.

**Reflexive monitoring: What did stakeholders to evaluate and monitor the implementation of the programme and adapt it as needed?**

This domain refers to the mechanisms through which stakeholders could monitor the implementation of the vaccination programme and its outcomes. The BNSSG programme had various mechanisms for reflexive monitoring including: The daily meetings of *the multi-disciplinary leadership team; national and local* vaccine uptake *data* and the qualitative data gathered by the *Communication, Insight and Engagement team* to inform local *decision-making;* empowering individuals at all levels to appraise the implementation of the programme, and use their *skills and expertise* to adapt it to enable successful implementation in their setting; and adapting the assessment of outcomes of the programme, against *financial costs* to accurately reflect the cost-savings associated with vaccine uptake in at risk populations.

**References**

1. Wang Y, Bye J, Bales K, Gurdasani D, Mehta A, Abba-Aji M, Stuckler D, McKee M. Understanding and neutralising covid-19 misinformation and disinformation. BMJ. 2022 Nov 22;379:e070331. doi: 10.1136/bmj-2022-070331.

2. Berrou I, Hamilton K, Cook C, Armour C, Hughes S, Hancock J, Quigg S, Hajinur H, Srivastava S, Kenward C, Ali A, Hobbs L, Milani E, Walsh N. Leaving No One Behind: Interventions and Outcomes of the COVID-19 Vaccine Maximising Uptake Programme. Vaccines (Basel). 2022 May 25;10(6):840. doi: 10.3390/vaccines10060840.

3. McGowan VJ, Bambra C. COVID-19 mortality and deprivation: pandemic, syndemic, and endemic health inequalities. Lancet Public Health. 2022 Nov;7(11):e966-e975. doi: 10.1016/S2468-2667(22)00223-7.

4. NHS England. Integrated care in your area. Integrated care systems and integrated care boards in England. 2022. [cited 16 December 2022]. Available from: <https://www.england.nhs.uk/integratedcare/integrated-care-in-your-area/#:~:text=Each%20ICS%20has%20an%20integrated,of%20health%20services%20in%20the>

5. Wolfe RM, Sharp LK. Anti-vaccinationists past and present. BMJ. 2002 Aug 24;325(7361):430-2. doi: 10.1136/bmj.325.7361.430.

6. NHS England (2020). NHS vaccine programme ‘turning point’ in battle against the pandemic. 2020. [cited 29 December 2022]. Available from: <https://www.england.nhs.uk/2020/12/nhs-vaccine-programme-turning-point-in-battle-against-the-pandemic/>

7. NHS. NHS vaccinations and when to have them. 2022. [cited 29 December 2022] Available from: <https://www.nhs.uk/conditions/vaccinations/nhs-vaccinations-and-when-to-have-them/>

8. Harnden A, Lim WS, Earnshaw A. COVID-19 vaccination programme: a central role for primary care. Br J Gen Pract. 2021 Jan 28;71(703):52-53. doi: 10.3399/bjgp21X714929.

9. Wilkinson E. Covid-19 vaccine outreach: "local knowledge, contacts, and credibility really, really matter". BMJ. 2021 Jun 18;373:n1547. doi: 10.1136/bmj.n1547.

10. May CR, Cummings A, Girling M, Bracher M, Mair FS, May CM, Murray E, Myall M, Rapley T, Finch T. Using Normalization Process Theory in feasibility studies and process evaluations of complex healthcare interventions: a systematic review. Implement Sci. 2018 Jun 7;13(1):80. doi: 10.1186/s13012-018-0758-1.

11. Hawe P, Shiell A, Riley T. Theorising interventions as events in systems. Am J Community Psychol. 2009 Jun;43(3-4):267-76. doi: 10.1007/s10464-009-9229-9.

12. Royal College of General Practitioners. Delivering Mass Vaccinations During COVID-19 A Logistical Guide for General Practice. 2020. [cited 22 December 2022]. Available from: <https://elearning.rcgp.org.uk/pluginfile.php/149506/mod_page/content/78/Mass%20Vaccination%20at%20a%20time%20of%20COVID%20V3.1.pdf>

13. Office for National Statistics. People living in deprived neighbourhoods—GOV.UK Ethnicity facts and figures. 2020. [cited 20 December 2022]. Available from: <https://www.ethnicity-facts-figures.service.gov.uk/uk-population-by-ethnicity/demographics/people-living-in-deprived-neighbourhoods/latest>

14. Sayegh L, Anthony W P & Perrewé P L. Managerial decision-making under crisis: The role of emotion in an intuitive decision process. Hum. Resour. Manag. Rev., 2004; 14(2), 179–199. <https://doi.org/10.1016/j.hrmr.2004.05.002>

15. Khatri N & Ng H A. The Role of Intuition in Strategic Decision Making. Human Relations, 2000; 53(1), 57–86. <https://doi.org/10.1177/0018726700531004>

16. Sharps MJ, Martin SS. "Mindless" decision making as a failure of contextual reasoning. J Psychol. 2002 May;136(3):272-82. doi: 10.1080/00223980209604155.
